# Identifying Enablers of Participant Engagement in Clinical Trials of Consumer Health Technologies: Qualitative Study of Influenza Home Testing

**DOI:** 10.2196/26869

**Published:** 2021-09-14

**Authors:** Spurthy Dharanikota, Cynthia M LeRouge, Victoria Lyon, Polina Durneva, Matthew Thompson

**Affiliations:** 1 Department of Information Systems and Business Analytics Florida International University Miami, FL United States; 2 Primary Care Innovation Lab Department of Family Medicine University of Washington Seattle, WA United States

**Keywords:** consumer health care technologies, CHTs, smartphone-supported home tests, Smart-HT, premarket clinical trials, trial engagement, at-home diagnostic testing, mobile phone

## Abstract

**Background:**

A rise in the recent trend of self-managing health using consumer health technologies highlights the importance of efficient and successful consumer health technology trials. Trials are particularly essential to support large-scale implementations of consumer health technologies, such as smartphone-supported home tests. However, trials are generally fraught with challenges, such as inadequate enrollment, lack of fidelity to interventions, and high dropout rates. Understanding the reasons underlying individuals’ participation in trials can inform the design and execution of future trials of smartphone-supported home tests.

**Objective:**

This study aims to identify the enablers of potential participants’ trial engagement for clinical trials of smartphone-supported home tests. We use influenza home testing as our instantiation of a consumer health technology subject to trial to investigate the dispositional and situational enablers that influenced trial engagement.

**Methods:**

We conducted semistructured interviews with 31 trial participants using purposive sampling to facilitate demographic diversity. The interviews included a discussion of participants’ personal characteristics and external factors that enabled their trial engagement with a smartphone-supported home test for influenza. We performed both deductive and inductive thematic analyses to analyze the interview transcripts and identify enabler themes.

**Results:**

Our thematic analyses revealed a structure of dispositional and situational enablers that enhanced trial engagement. Situationally, clinical affiliation, personal advice, promotional recruitment strategies, financial incentives, and insurance status influenced trial engagement. In addition, digital health literacy, motivation to advance medical research, personal innovativeness, altruism, curiosity, positive attitude, and potential to minimize doctors’ visits were identified as the dispositional enablers for trial engagement in our study.

**Conclusions:**

We organized the identified themes for dispositional and situational enablers of trial engagement with a smartphone-supported home test into a research framework that can guide future research as well as the trial design and execution of smartphone-supported home tests. We suggest several trial design and engagement strategies to enhance the financial and scientific viability of these trials that pave the way for advancements in patient care. Furthermore, our study also offers practical strategies to trial organizers to enhance participants’ enrollment and engagement in clinical trials of these home tests.

## Introduction

### Background

Consumer health care technologies (CHTs) that could potentially transform the health care industry require proper assessment and evaluation before large-scale use. In 2013, the US Food and Drug Administration (FDA) issued guidelines named *Mobile Medical Applications Guidance for Industry and Food and Drug Administration Staff* to regulate a rapidly growing number of mobile consumer health apps [1]. Furthermore, the FDA defined *mobile medical applications* as medical devices that are mobile apps or as an accessory to regulated medical devices that require FDA approval [1]. Personal health information is increasingly intertwined with clinical applications. In addition, CHTs are increasingly being looked at as a means to support the effective execution of home-based medical diagnostic tests [2-4] and medical procedure preparation kits (eg, colonoscopy preparation) [5]. As a result, an increasing number of CHTs will require assessment and evaluation (eg, clinical and premarket trials). Premarket trials are often an integral part of health care product development, even when regulatory assessments are not formally required. Developers and vendors may seek to pretest CHTs with target users for usability, feasibility, and economic viability [6]. Specifically, they provide ways to evaluate the CHT’s impact on health outcomes and assess efficacy and safety before releasing it to the general population [7]. Trials can also provide opportunities for refinements to improve CHTs before their availability for commercial use [8]. Overall, trial engagement involving CHT is an interesting user behavior that deserves more attention in research to facilitate the assessment of health care innovations for the suitability for general use.

We define a potential trial participant as someone willing to try the technology before it becomes available for public and wider-scale use. Trial engagement refers to a potential trial participant successfully completing a trial. Research generally reports inadequate trial engagement as a significant challenge to CHT trial success [9]. For example, potential trial participants may perceive trial participation as a risky activity and may not be willing to enroll [10]. In addition, potential trial participants may determine that incentives or compensation are inadequate compared with their perceived risk of participating in the trial [11]. Even after enrollment, the participants’ lack of engagement in trial activities and high dropout rates can impede trial success [12]. In the context of CHT, studies show that inadequate enrollment and engagement can be very costly for CHT manufacturers and researchers involved [13-15]. By and large, trial engagement is crucial for determining the validity, feasibility, and success of CHT clinical trials [16-18].

Unfortunately, there is a lack of research to provide guidance regarding people’s perceptions and attitudes toward engaging in CHT trials. Current research that addresses engagement in clinical trials focuses primarily on evaluating attitudes toward traditional in-person trials of pharmaceutical interventions such as pharmaceutical trials [19] and randomized control trials for interventions and care processes related to serious health conditions such as cancer [20-22], cardiovascular disease [23,24], and lung disease [25].

Indeed, there is a shortage of behavioral research related to trials that evaluate engagement in CHT trials involved in the clinical or self-care process [26]. Within the realm of CHT, health apps offer tools, procedures, and communications to support mobile health care practices [27,28]. Moreover, very few studies in the medical informatics literature have explored clinical trials involving health apps [29], and few of the existing studies aim to identify enablers for health apps. Consumer perceptions or beliefs about the trial attributes that influence clinical trial engagement are known as enablers [30]. One notable exception in the medical informatics literature includes a 2-pronged research study, which proposed a recruitment framework for eHealth clinical trials using cost-effective and time-efficient trial recruitment strategies [18,31]. However, the study did not consider the attitudes of potential trial participants toward the trial. Another notable exception is a study that examined the perceptions and experiences of women engaging in a digital technology–based clinical trial in the context of physical activity interventions [32]. The study identified critical factors that enabled participants’ continued engagement in physical activity after the trial period but did not investigate the enabling factors for their trial participation. In a different study context, Cohen et al [33] tried to understand patient compliance during 2 digital trials involving 2 pathologies, Parkinson disease and Huntington disease, each lasting 6 months. The study measured patient compliance metrics, namely, daily app-based medication reporting during the 6-month trials; however, it did not attempt to understand the factors that enabled participants’ trial engagement.

Identifying engagement enablers can inform future trial design and execution, thereby enhancing the scientific and financial feasibility of CHT trials. Among the increasing technological capabilities associated with CHT innovation is using a smartphone to support home-based diagnostic tests, referred to as smartphone-supported home tests (Smart-HT). This study focuses on trial engagement in the context of CHT used for home-based diagnostics or Smart-HT (ie, diagnostic tests that a user can carry out with the support of a smartphone) [34-37]. Smart-HT usually requires FDA approval and has growing market potential and patient interest. Smart-HT comprises a diagnostic testing kit and a software component (eg, a mobile app providing one or more of the following features: instructions, education about the test or health situation, an indication of results, and results messaging). The user is required to simultaneously perform the necessary physical procedures to complete the test and understand the software component to use Smart-HT to achieve the desired outcome—diagnosis and enablement for the next steps in care.

The growing interest in at-home diagnostics, combined with their potential implications for future pandemic preparedness, merits the focused study of Smart-HT. The at-home diagnostic market is predicted to value >US $6 billion by the end of 2027; the market is anticipated to have a compound annual growth rate of 3.98% during the forecast period of 2020 and 2027 [38]. A recent survey (the 2018 Deloitte Center for Health Solutions) indicated that most people are interested in engaging with new care channels, such as at-home diagnostic testing. Overall, 51% of respondents reported being comfortable using at-home tests for their current health concerns and identifying potential future health issues. Furthermore, 44% of respondents reported being comfortable using mobile apps connected to at-home diagnostic tests to track and monitor their health trends [39].

### Objective

The purpose of this study is to identify the enablers of potential participants’ trial engagement in clinical trials of CHT, in particular, Smart-HT trials. We use flu@home, a Smart-HT in trial that comprises a test kit and a mobile app to facilitate home-based testing for the diagnosis of influenza, as our instantiation of CHT to address the following research question: What enablers influence trial engagement for Smart-HT?

The paper is structured as follows. First, we discuss the Smart-HT context of the study (flu@home pilot) used to inform the enablers of Smart-HT trial engagement. We provide details on the interview phase of the pilot, where qualitative data were collected and analyzed to inform the results of this study. Second, we present our results in the form of a Smart-HT trial engagement research framework based on our evidence and provide a discussion of the identified enablers for Smart-HT trial engagement. Finally, we discuss the implications of this study for trial organizers and researchers.

## Methods

### Smart-HT Context: flu@home

This study leverages the context of influenza (or *flu*) using an instantiation of Smart-HT called flu@home, which has the potential to allow individuals to self-diagnose influenza. Influenza, a contagious respiratory infection caused by influenza viruses, is a serious global health threat with an estimated 1 billion cases each year, of which 18% of people die worldwide [40]. Influenza imposes a substantial economic burden, including health care costs and productivity losses, accounting for US $87 billion in the United States alone [41]. Infectious diseases, including influenza, call attention to the need for speedy diagnosis and patient treatment or isolation [3]. In addition, the drugs available for treating influenza are most effective when used within 48 hours of identifying symptoms [42]. Smart-HT offers an expedient and effective way of diagnosing influenza at home without exposing others to the virus (eg, at a physicians’s office).

**Figure 1:**
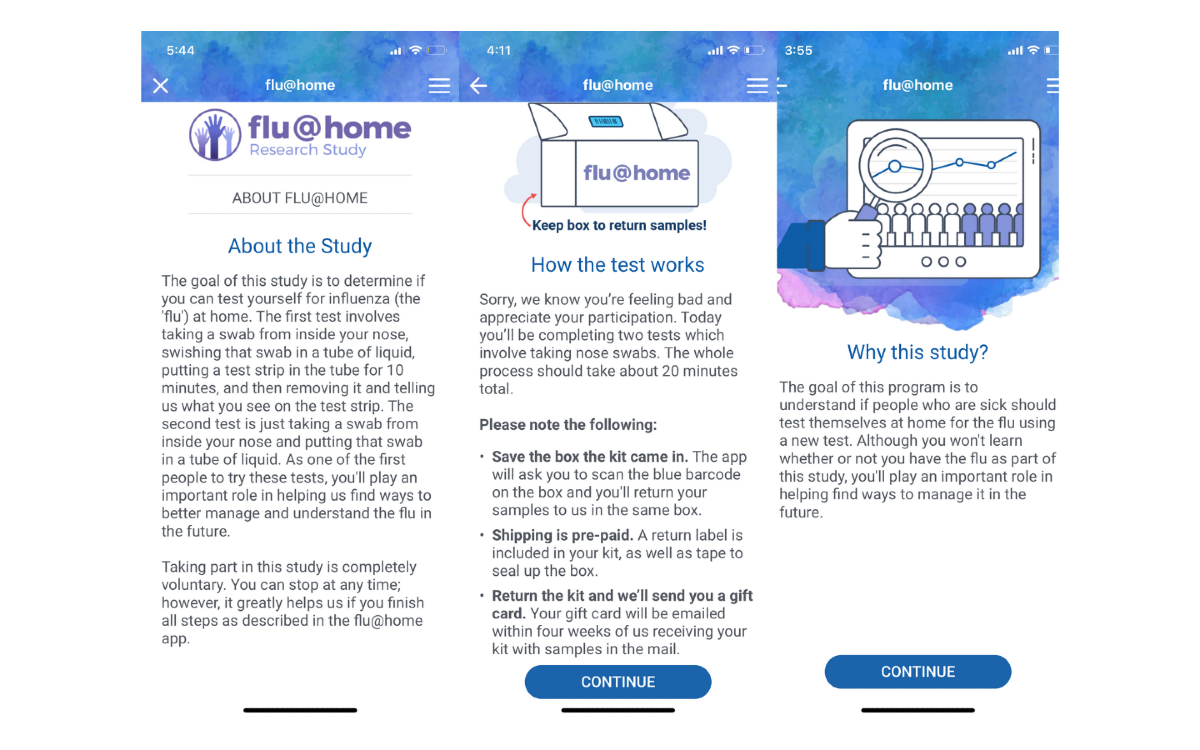
Screenshots of flu@home app.

The Smart-HT in this study, flu@home, comprises two components: a test kit (adapted from the Quidel QuickVue Influenza A+B test) and a mobile app to facilitate testing (Figure 1). The at-home rapid diagnostic testing kit includes materials for an individual to self-test for influenza using a low nasal swab. The testing involves taking a sample from inside one’s nose and processing it using a lateral flow assay (Quidel Corporation). The mobile app was available for participants to download on the iOS platform for use on smartphones and tablets. Multimedia Appendix 1 contains detailed information about the technical details associated with the flu@home mobile app.

**Figure 2:**
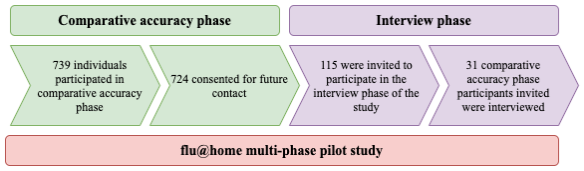
Phases of flu@home pilot study.

The flu@home pilot study included two phases: the comparative accuracy phase and the interview phase (Figure 2). The study design was approved by the University of Washington institutional review board STUDY00007627.

During the comparative accuracy phase, participants engaged with flu@home. The inclusion criteria for the comparative accuracy phase of the flu@home pilot study involved eligible participants who were aged ≥18 years, spoke English, had an iPhone or iPad, and had an influenza-like illness, defined as the presence of a cough and at least one or more of the following symptoms: fever, chills or sweats, muscle or body aches, or feeling tired or more tired than usual. Two videos were provided to participants as part of this study: one for recruitment purposes (study overview) and the other for providing step-by-step instructions to engage in the trial (Multimedia Appendices 2 and 3 contain the videos). Trial participants (N=739) completed the following workflow: (1) downloaded the flu@home app to enroll in the trial and order a test kit, (2) completed the consent form and a survey about their symptoms and exposure risk, (3) received a test kit by mail and followed the app instructions to conduct the rapid diagnostic test, and (4) shipped a second nasal swab sample to the research laboratory for reference standard testing. Of the 739 individuals who participated in the comparative accuracy phase of the flu@home trial, 97.9% (724/739) trial participants consented to be contacted for further phases of research.

During the interview phase, we conducted semistructured interviews with a sample of individuals who completed the comparative accuracy phase. We used phenomenology to guide this phase to construct a rich understanding of participants’ experiences with flu@home and identify the enablers of Smart-HT trial engagement [43]. Phenomenology, described as the science of phenomena, explores human experience to elicit meanings for individuals through the analysis of their experiences and perceptions [44]. In line with this paradigm, we conducted in-depth interviews that covered experiences and perception questions related to (1) personal characteristics that enabled participants to complete flu@home trials; (2) attitudes toward health and medical research; (3) perceptions toward flu@home, mobile apps, and the overall trial; and (4) factors that enabled their Smart-HT trial engagement.

To fulfill the purposes of this study, we reported results from the interview phase related to understanding the enablers of potential participants’ Smart-HT trial engagement. In the following sections, we further describe the data collection and data analysis procedures pertinent to the interview phase.

### Data Collection

To meet the eligibility (inclusion) criteria for the interviews, individuals must have participated in the comparative accuracy phase of the pilot and consented in the flu@home app to be contacted for future study efforts. However, participation in the interview phase was not dependent on individuals completing the final steps in the flu@home workflow (ie, it is possible that some participants never mailed their sample to the laboratory; however, they would still be eligible if they opted in for future contact in the app). The flu@home test results were deidentified, so we were unable to determine how many eligible participants may not have performed the final step of mailing back their test results for analysis.

We consulted the literature to determine the total number of participants for the interviews. Data saturation, the point at which additional data collection no longer generates a new understanding [45], is the most common guiding principle for determining the total number of participants in qualitative research [46]. Prior interview study designs reached saturation in as few as 10-40 interviews [47-52]. In considering precedence and study design, we determined a minimum of 20 trial participants to be an adequate and appropriate target number of interviews.

Participants were invited to participate in the interviews via email and offered a US $25 gift card for completing the interview. To ensure diversity in the interviewee sample, we recruited participants in three waves. We followed purposive sampling to maintain diversity in the representation of trial participants in terms of age, race, and geographic location. First, we sorted the trial participants into age groups (18-24 years, 25-34 years, 35-44 years, 45-64 years, and ≥65 years) and randomly selected participants from each age group to send interview invitations. In the two subsequent waves of recruitment, we adjusted the proportion of participants recruited from each age group to ensure sample representation from all age groups. Attempts to fulfill purposeful sampling resulted in 115 invitations to participate and 31 actual interviews. Table 1 details the demographics of the trial participants who were invited and interviewed for the study.

**Table 1:** Demographics of interview participants in the study.

Characteristics			Invited to participate in study		Completed the interview
Total, n (%)			115 (100)		31 (100)
**Age (years), n (%)**					
	18-24	12 (10.4)		3 (10)	
	25-34	34 (29.6)		6 (19)	
	35-44	38 (33)		11 (36)	
	45-64	21 (18.3)		8 (26)	
	≥65	10 (8.7)		3 (10)	
**Ethnicity, n (%)**					
	White individuals	78 (67.8)		21 (68)	
	Black or African American	10 (8.7)		6 (19)	
	Asian	8 (7)		0 (0)	
	Native Hawaiian or Other Pacific Islander	1 (0.9)		1 (3)	
	American Indian or Alaska Native	16 (13.9)		1 (3)	
	N/A^a^, other, or prefer not to say	2 (1.7)		2 (6)	
**Geographic representation,** **n (%)**					
	West	43 (37.4)		14 (45)	
	Midwest	21 (18.3)		5 (16)	
	Southwest	2 (1.7)		1 (3)	
	Northeast	32 (27.8)		4 (13)	
	Southeast	17 (14.8)		7 (23)	

^a^N/A: not applicable.

Semistructured interviews were conducted via the web-based video conferencing tool Zoom from August 12, 2019, to December 19, 2019. Zoom has been an effective means of data collection in various health-related studies [53]. Participants had the option of being on or off camera. Each interview lasted between 40 and 60 minutes. Of the 3 research team members (in a rotating fashion), 2 were present during the interviews, providing direct experience with the actual interviews to the 3 team members subsequently engaged in the analysis process. Of the 2 team members participating in the interview, 1 served as the lead interviewer, whereas the other served the role of *active listener* and notetaker. The *active listener* was invited to ask any follow-up questions needed for clarification during the interview. The 3 team members alternated in serving the primary interviewer and listener roles for each interview. The 2 team members participating in the interview debriefed immediately after each interview to review highlights relevant to the study, establishing early key points to consider for coding purposes. Interviews were recorded and transcribed, and all personal identifiers were removed before analysis. We uploaded deidentified interview transcripts to Dedoose version 7.0.23, a software for qualitative data analysis.

### Data Analysis

**Figure 3:**
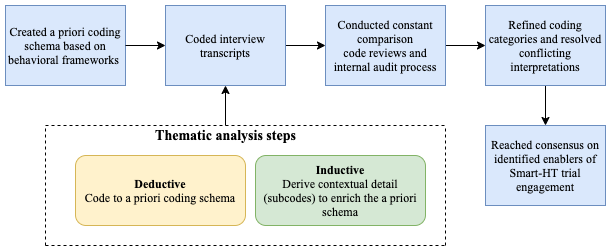
The qualitative thematic analysis process that guided the deductive and inductive coding of the interview transcripts. Smart-HT: smartphone-supported home tests.

We conducted a thematic analysis [54] to code the deidentified transcripts. Thematic analysis is defined as a method for identifying, analyzing, organizing, describing, and reporting themes within a data set [55]. To ensure the validity and reliability of the thematic analysis results, we followed the Lincoln and Guba [50] criteria for conducting qualitative research. Specifically, we established confirmability, dependability, and credibility through procedures such as researcher triangulation, code reviews, expert feedback, and resolution meetings [50,56]. We took a hybrid approach to thematic analysis by including both deductive and inductive coding [57]. Figure 3 illustrates this process.

We developed the a priori coding schema based on widely used behavioral frameworks that explain an individual’s behaviors and actions [58-61] and categorized the factors into dispositional and situational enablers. Specifically, we relied on attribution theory, the digital health engagement model proposed by O’Connor et al [61], and the technology acceptance model [60] to identify generalized enabler constructs that align with our context to include in our a priori coding schema. On the basis of this foundation, our resulting a priori coding schema recognized the possibility of personal, perceptional, and situational factors. Situational enablers referred to external events or environmental factors that contributed to participants’ Smart-HT trial engagement [58,59]. Personal enablers related to an individual’s abilities, traits, and beliefs, whereas perceptional enablers referred to their perceptions that enabled their Smart-HT trial engagement. To further refine our schema and acknowledge relationships in existing research, we decided to group the personal and perceptional enablers we find in the data as *dispositional*.

**Figure 4:**
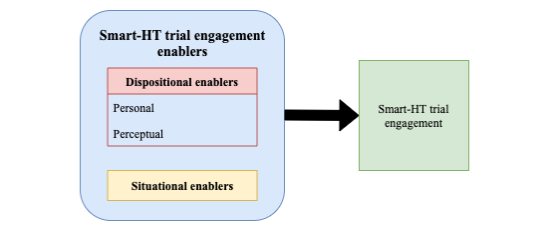
A priori coding schema for thematic analysis. Smart-HT: smartphone-supported home tests.

Dispositional enablers referred to trial participants’ behaviors and perceptions, whereas situational enablers were independent of participants' behavior. We used the skeletal a priori coding schema (Figure 4) as a starting point for the thematic analysis. Using the schema facilitated an initial, agreed-upon conceptual basis for the research team to begin analysis and then determine the operational definitions for each identified variable based on the schema [62,63].

We started our thematic analysis with a deductive approach [57]. During this analysis step, 2 of our team members independently coded the interview transcripts using the a priori coding schema (Figure 4). We were also open to additional high-level constructs emerging from our study that would extend the a priori schema. To capture key content within each a priori theme (ie, parent code), we extended and enriched the a priori schema codes (ie, parent codes) with inductively identified subcodes (ie, child codes). Explicitly, as we encountered new content during the coding process, we updated our coding schema with additional subcodes to further define the general themes in the a priori schema to the Smart-HT context. These practices further defined and specified associated constructs in the a priori coding schema and continued until thematic saturation of the Smart-HT trial enablers was reached.

We used the constant comparison method of analysis [63,64] to refine and triangulate the coding. The constant comparison procedure included 2 coders and an internal auditor serving. A research team member with >17 years of qualitative research methods expertise, familiar with the study constructs and context but not engaged in the detailed coding process, served as our internal auditor to ensure expertise, understanding, and independence from the specific assignment of quotes to codes and initial code labels. The team held recurring meetings to compare initial coding determinations to ensure agreement on high-level codes and ratify the addition of subcodes (child codes) supporting the constructs in the initial coding schema. The team also refined the code labeling (names of codes) as needed to ensure understandable terms and themes. The ratification of subcodes included a general review of supporting quotes to support discussion points.

Although various studies finalize coding on this consensus [65], we chose to extend our rigor to support our findings further. Specifically, as a final step, our internal auditor also reviewed the holistic structure, labeling, and syntax of the final reconciled schema and performed a code review of 100% of the coded quotes to ensure alignment with the final coding structure. Any identified issues were brought back to the collective team for final disposition. As noted in prior literature, a feedback process, such as this internal audit process, can serve as a crucial *reader resonation* strategy to further enhance the validity of qualitative findings [66].

There were no noted major conceptual differences in the constant comparison and final internal review process. Reconciliation and arbitration were primarily focused on combining some subcodes or refining the labels and definitions of added codes. In particular, the team sought to refine the labels, where appropriate, to terms used in generally related research. In completing the process, there were no exceptions to reaching consensus, and, thus, code reviews and resolution meetings established the confirmability and dependability of the findings. We present our results as a research framework for enablers of Smart-HT trial engagement in the following section.

## Results

### Overview of Results

**Figure 5:**
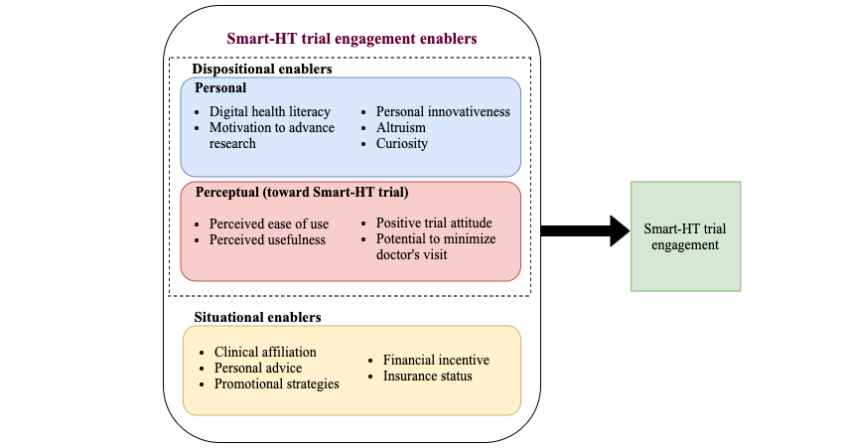
Evidence-based smartphone-supported home tests trial engagement research framework. Smart-HT: smartphone-supported home tests.

We synthesized our results into a research framework (Figure 5) that presents pertinent dispositional and situational enablers for Smart-HT trial engagement. This section details our thematic analysis findings under each of the broader enabler themes (dispositional and situational). We include evidence trace tables providing a representative participant quote to demonstrate the study’s identified themes, as found in the data in Multimedia Appendix 4. Tables S1, S2, and S3 provided in Multimedia Appendix 4 present the evidence trace table for personal enablers and perceptional and situational enablers, respectively. The research team collectively selected the quotes from the multiple options coded as both a good conceptual fit and easy for the reader to understand outside of providing extensive interview text and context.

### Dispositional Enablers

Our results supported characterizing dispositional enablers into personal and perceptional.

#### Personal Enablers

Our results identified the following personal enablers for Smart-HT trial engagement: digital health literacy, motivation to advance medical research, innovativeness, curiosity, and altruism. Table S1 in Multimedia Appendix 4 presents the evidence trace table for personal enablers identified in the study.

In general, the interviewees in this study were digital health literate. Interviewees commonly expressed their ability to seek, find, understand, and apply health information from a wide range of web-based sources such as Google, health care websites, and WebMD, among others. Moreover, some interviewees even noted their familiarity with using wearables (eg, Apple Watch) and telemedicine for taking care of their health, which evidenced their adequate degree of *digital health literacy.*

Our inductive thematic analysis emphasized *motivation to advance medical research* as a prominent enabling factor in trial engagement for Smart-HT. The data revealed that participants hoped that their feedback after the flu@home trial might benefit vaccine development in the future. An interviewee explained that offering feedback and data that further medical research is vital for the greater good.

Remarkably, nearly all the interview participants acknowledged that they were *inherently innovative*. Their perception of Smart-HT as an innovative and transformative technology seemingly encouraged them to engage in the trial. Our analysis further identified *curiosity* in a relatively new technology (ie, a Smart-HT) as a personal enabler for individuals to engage in the trial. In addition to being curious about the trial and technology, interviewees indicated how their *altruistic notions* encouraged them to participate in the trial.

#### Perceptual Enablers (Toward Smart-HT Trial)

Our analysis revealed the importance of trial participants’ perceptions of Smart-HT in their trial engagement for Smart-HT. The perceptual enablers identified in the study (Table S2 in Multimedia Appendix 4) include ease of use, usefulness, positive attitude, and potential to minimize physician visits.

The interviewees perceived the overall Smart-HT trial to be easy to follow and self-explanatory. For example, many described their use of nasal swabs to self-test for influenza as an *easy process.* Participants also stressed how the test kit packaging with vials to hold to swabs made the whole Smart-HT trial engagement process simpler. Interviewees also commented on the mobile app’s interface that guided them with the instructions required to complete the trial as not complicated and that it served the intended purpose.

In addition, *the perceived usefulness* of the Smart-HT and the value of their participation in advancing research seemed to have encouraged interviewees’ Smart-HT trial engagement. Interviewees perceived their trial engagement with flu@home as a *useful* exercise contributing to public health management in enhancing the Smart-HT functionalities for large-scale commercial use. Participants also indicated that flu@home was *useful* in reducing the spread of disease in the future.

Overall, the interviewees cited a *positive attitude* toward home-based testing and their trial engagement for the Smart-HT (*positive trial attitude*). Many expressed optimism toward Smart-HT and found it to be a promising and innovative technology with potential uses. Excitement at being offered to be a part of the Smart-HT trial was also a common theme among the interviews. Several interviewees reported their interest in engaging in a trial for a promising Smart-HT that could minimize traditional physician's visits for illness diagnosis. In addition, most interviewees explained how getting a physician’s appointment when most needed could be a hassle that they would readily forego for a Smart-HT.

### Situational Enablers

Situational enablers referred to external events or environmental features that enabled trial engagement for the Smart-HT. Our analysis revealed clinical affiliation, personal advice, promotion strategies, financial incentives, and insurance status as situational enablers for Smart-HT trial engagement. We present a representative quote for each of the situational enablers identified in this study (Table S3 in Multimedia Appendix 4).

*Clinical affiliations* of the health care entities associated with the trial prominently influenced interviewees’ willingness to engage in the Smart-HT trial. Several interviewees explained that the reputable institutions associated with flu@home’s development and the trial execution enhanced their perceived trust in the trial, which resulted in their trial engagement.

Furthermore, interviewees expressed their propensity to trust *personal advice* or recommendations from friends and family members to participate in these Smart-HT trials. A few interviewees shared details about who recommended them to the Smart-HT study. The list of personal recommenders identified included friends, spouses, and family members.

Considering the voluntary nature of trial participation, we identified the salience of effective *promotion and recruitment strategies* in enabling individuals’ engagement in the Smart-HT trial. Interviewees detailed how they found out about the Smart-HT study on the web and described how web-based advertising could be a helpful recruitment mode. In addition, the interviewees offered meaningful insights into preferred future web-based recruitment and promotion strategies. From our analysis, social media emerged as a popular recruitment pathway to achieve the desired trial participation. The data also included references to a web-based forum or an email list where people could volunteer for these trials.

The *financial incentive* that was offered in the form of a gift card provided by the research study at completion notably incentivized and compensated the participants to engage in the Smart-HT trial. An interviewee also emphasized how *lack of insurance* coupled with symptoms and incentives encouraged Smart-HT trial engagement.

## Discussion

### Overview

In this qualitative study, we explored the enablers of trial engagement with a home-based diagnostic supported by a CHT, referred to as Smart-HT. We identified dispositional and situational enablers of Smart-HT trial engagement that can inform future Smart-HT trial design and execution. In the following sections, we discuss these enablers, along with recommendations for trial organizers and researchers.

In reviewing the following discussion of enablers, it is worth considering that many CHTs in various health care contexts involve a software component and are rooted in a traditional health care service offering. Additional forms of CHTs may elicit similar trial engagement responses as those who responded to the Smart-HT context. Therefore, it is plausible that the identified Smart-HT dispositional and situational enablers we discuss may apply to a majority of CHT trials, in general, and merit testing in other CHT contexts.

### Dispositional Enablers

#### Personal Enablers

With clinical trials facing challenges in the form of low retention and high dropout rates of trial participants, our results point to the need for increased attention on personal enablers in future Smart-HT trial recruitment efforts. In alignment with prior studies on digital health technologies [67,68], individuals with digital health literacy (eg, prior experience with digital health tools) generally tended to have a positive experience with Smart-HT. In our study, individuals generally reported to be digitally health literate and did not encounter any major issues during the Smart-HT trial. Experience with accessing health care on the web seemed to have enabled participants to engage with Smart-HT in the trial. Digital health literacy of potential trial participants could inform their Smart-HT trial engagement behaviors. Therefore, as part of the participant recruitment efforts, trial organizers may want to develop screening criteria to recruit individuals with proficient digital health literacy among the target appropriate populations. To reach those with no relevant digital health tool experience, we suggest that the trial organizers offer appropriate training and general promotion concerning Smart-HT use to enhance Smart-HT trial engagement.

Smart-HT trial engagement was also enabled by the trial participants’ willingness to play a role in advancing medical research. These results are in line with a survey conducted on public perceptions in the United States toward clinical trials in which 86% of the sample noted that they would participate in clinical trials for advancing scientific research [69]. In the future, researchers should make use of appropriate advertising channels to communicate the potential value of the Smart-HT trial in advancing medical research to prospective trial participants. An increase in the perceived value of the trial might further motivate prospective participants’ Smart-HT trial engagement intentions.

Personal attributes, such as inherent innovativeness, curiosity, and altruistic notions, enabled participants' Smart-HT trial engagement. These findings offer practical implications for Smart-HT trial designers and trial organizing entities, as they inform successful trial participant recruitment in future trials. For instance, researchers in future trials could extend a short survey as part of the screening criteria to measure personal innovativeness [70] and curiosity [71] as part of the Smart-HT trial recruitment processes. Potential trial participants who are relatively more innovative and curious about the Smart-HT (in the trial) could then be screened, thereby enhancing the Smart-HT trial engagement and success. We also propound that future trial designers could highlight their choice of individuals who explore and are curious about new technologies in their recruitment campaigns. For instance, clear recruitment messages such as “Do you want to try a new and transformational healthcare technology?” and “Are you interested in contributing to your community” could further encourage potential trial participants’ Smart-HT trial engagement.

#### Perceptual Enablers (Toward Smart-HT Trial)

The successful design of the accompanying mobile app enabled the overall success of the Smart-HT trial. The mobile app instructions were perceived to be self-explanatory and easy to use and enabled the participants’ Smart-HT trial engagement**.** Easily understood and navigable mobile app interfaces with clear and step-by-step instructions (which in this case included videos) are critical to ensuring that trial participants complete the trial successfully and do not withdraw from the study because of frustration with the test procedures or technology. This finding further strengthens this study's contribution as it points to the potential of Smart-HT. Namely, the addition of a technology component to a trial or even an existing home diagnostic test to comprise a Smart-HT has the potential to alleviate some of the concerns of errors or process misunderstanding surrounding home diagnostic self-testing in the absence of clinical supervision. The addition of technology to facilitate procedural success can further inform future trials. Future researchers could examine whether trials involving tests and home-based preparation procedures (eg, blood test) combined with the easy-to-use app would have better trial engagement outcomes than trials involving diagnostic tests accompanied by no apps or complicated apps.

Our findings suggest a few considerations regarding the specific design of apps to support trial procedures. First, trial mobile app developers could ensure that the trial’s aims and objectives are adequately communicated through the app pages to enhance the Smart-HT trial engagement. Second, the app should contain a frequently asked questions section that addresses the common concerns or risks surrounding the trial. Third, the app should provide an option to call a research coordinator to answer any questions that may arise during the trial. Fourth, interactive videos demonstrating the step-by-step study procedures (eg, nasal swab process) may stimulate and inspire potential trial participants to engage. All these design elements may reduce trial drop-off.

In line with our findings, prior research on the trialability of new technologies has shown that a positive attitude [72,73] toward the technology can ease concerns surrounding a trial of relatively new technology like Smart-HT. Removing this concern eliminates a barrier to motivating individuals to engage with Smart-HT. Furthermore, Smart-HT trial engagement was perceived to contribute to public health management in our study. These findings have significant implications for trial organizers and mobile app developers who could highlight how a specific trial could advance and benefit public health in addition to advancing medical research. Consequently, educating potential trial participants about positive trial consequences may manifest in a positive attitude toward the Smart-HT and the overall trial, encouraging the Smart-HT trial engagement.

Another interesting perceptional enabler theme that emerged from our analysis was the perceived potential of the Smart-HT to minimize a physician’s visit. Smart-HT offers a fundamental change from traditional health care encounters for diagnostic testing. The potential role of Smart-HT in reducing the need to seek face-to-face appointments encouraged the interviewees to engage in the trial. Therefore, Smart-HT trial engagement may be enhanced if potential trial participants are convinced that the Smart-HT is transformational and offers solutions to the existing problems they face in the health care system (eg, access to testing and getting physician’s appointments). Researchers in future trials should inform potential trial participants about the possible innovations and improvements that may be solved by the Smart-HT in trial.

### Situational Enablers

Awareness of the trial’s clinical affiliations enabled Smart-HT trial engagement in our study. The brand reputation associated with these health care and research institutions encouraged potential participants to trust the trial as legitimate and, thus, influenced their trial engagement. These findings are in line with a survey conducted on the perceptions toward clinical trials [69]. For example, 38% of the nationwide sample cited a lack of trust in trials as one of the primary reasons for not considering clinical trial participation. In addition, 91% of the same sample said that the competence and reputation of the trial conducting institutions play an essential role in their decision to participate in a trial. These findings could potentially inform future trial designers and mobile app developers. For example, future recruitment strategies could include detailed descriptions of the trial organizing institutions’ previous research breakthroughs and research quality (in terms of patents or publications) that could persuade potential trial participants to perceive the trial as legitimate and to engage in it.

Most of the trial participants heard about the Smart-HT trial in our study from social media. Our analysis affirmed that web-based advertising and recruitment strategies could be effective modes of potential participant recruitment. Web-based medical forums where potential trial participants could volunteer to engage in an advertised trial could offer a potential future mode of participant recruitment for Smart-HT trials. Sending Smart-HT trial invitations using emails to a list of potential trial participants could also enable the Smart-HT trial engagement. Therefore, Smart-HT trial organizers should dedicate significant efforts toward web-based recruitment using available channels such as web-based medical forums, email lists, health care blogs, and social media [74,75].

Personal recommendations to engage in the Smart-HT trial (in the form of referrals from friends and family) and the financial incentive offered for participating in the trial seemed to have motivated some interviewees’ engagement. In the future, mobile app developers could add a functionality, allowing one to invite others to sign up for the trial through the app itself. Many interviewees affirmed financial incentives as a critical factor that influenced their Smart-HT trial engagement. Although considering the perceived criticality of financial incentives, there may be value in offering additional incentives for referring other potential participants to the Smart-HT trial. Referral promotions could solve the issues of inadequate participant enrollment and retention rates.

Some participants attributed their trial engagement for the Smart-HT to their lack of health insurance. This study highlights the opportunity to engage participants in Smart-HT clinical trials to improve their access to needed health care. We also identified practical issues for trial designers in terms of trial advertising and recruitment efforts. One of the interviewees explained how the absence of trial participation costs encouraged her to enroll in the Smart-HT trial. Therefore, emphasizing that the Smart-HT trial is free and does not require health insurance in the advertisements and recruitment messages may increase engagement. These techniques could draw the attention of potential trial participants who may assume that they are ineligible for trial as they lack health insurance. Simultaneously, we caution the Smart-HT trial organizers that it would be unethical to coerce potential trial participants to sign up for a clinical study they would otherwise be reluctant to do.

Overall, our evidence-based research framework (Figure 5) highlights the cruciality of the trial participants’ personalities, beliefs, and perceptions as drivers of their Smart-HT trial engagement. Our analysis and the resulting framework may help Smart-HT researchers and trial organizers gain valuable insight into the phenomenon of trial engagement. We also encourage future research studies that extend our framework by examining the relative significance of the enablers presented using quantitative research methods.

### Limitations

We focused on one example of CHT, Smart-HT, an instantiation of flu@home to identify the dispositional and situational enablers of trial engagement in our study. Although we feel that our results may carry to other CHT trial situations, we did not test other CHT contexts. Therefore, this choice may limit the generalizability of the study’s findings. Specifically, trial participants for CHTs that differ from Smart-HT may elicit a different or an extended set of their trial engagement enablers. Although we expect most of the identified dispositional enablers to influence trial engagement for other CHTs and Smart-HTs, there could be additional situational enablers relevant to the functionality and context of the specific technology used. We encourage future research to investigate and enrich the framework in other contexts such as mobile health apps and Smart-HT for other health conditions and clinical trials. Our study focuses on offering an evidence-based Smart-HT trial engagement framework for Smart-HT that requires FDA approval. Future research is required to investigate the enablers for trial engagement for other health and wellness technologies that may not formally require FDA approval or undergo approval through different FDA routes for devices. The need for approval and situation influences resulting in social desirability response bias might have minimally influenced the qualitative study’s findings [76]. We encourage future studies to empirically test and extend our evidence-based Smart-HT trial engagement framework using quantitative research methods such as surveys.

### Conclusions

Premarket clinical trials play a crucial role in evaluating and assessing medical devices and technologies for effectiveness and safety before their approval and release to the general population. However, inadequate trial engagement remains an issue that leads to failure in CHT trials. In the field of CHT, there is a dearth of research exploring the factors leading to CHT trial engagement. This exploratory qualitative study reports enablers of trial engagement for Smart-HT, a promising form of CHT, using an instantiation to diagnose influenza, namely, flu@home.

Our study adds to the understanding of people’s perceptions toward Smart-HT trials by delineating an organized series of enablers that can impact the decision to participate in a Smart-HT trial. Clarification on these dispositional and situational enablers contributes to behavioral research associated with Smart-HT trials and can inform future Smart-HT trial design and engagement strategies. In addition, our study offers practical implications for trial organizers in the form of trial promotional and recruitment efforts. An important example is developing screening criteria to recruit innovative, curious, and altruistic individuals with proficient digital health literacy among the target population. Our study also confirms the benefit of creating relevant promotion materials that showcase Smart-HT’s usefulness, ease of use, benefits, trial organizer’s reputation and clinical affiliations, the trial’s role in advancing medical research, and finally, Smart-HT’s role in solving existing health care issues. Understanding and accounting for a series of internal and external enablers when targeting potential trial participants can address the issue of inadequate Smart-HT trial engagement, thereby leading to effective evaluation of health technologies and timely release of them to the general population.

## Appendix

Multimedia Appendix 1 The flu@home mobile app.

Multimedia Appendix 2 Trial recruitment videos—demo of flu@home.

Multimedia Appendix 3 Trial recruitment videos—overview of flu@home research study.

Multimedia Appendix 4 Evidence trace tables.

## Abbreviations

CHT: consumer health care technologies
FDA: US Food and Drug Administration
Smart-HT: smartphone-supported home tests
