# Supplementary material for: Identifying Enablers of Participant Engagement in Clinical Trials of Consumer Health Technologies: Qualitative Study of Influenza Home Testing
Source: J Med Internet Res. 2021 Sep 14;23(9):e26869. doi: 10.2196/26869 (PMC8479603; doi:10.2196/26869)
Supplement: Multimedia Appendix 1 [file jmir_v23i9e26869_app1.docx]

**Multimedia Appendix 1. flu@home Mobile Application**

flu@home is an iOS mobile application that served multiple functions in conducting the trial, such as screening participants for eligibility, acquiring consent electronically, and providing step-by-step instructions in a detailed manner for conducting a lateral flow test. The app also has instructions on how to return the swabs to the research team.

Audere is a Seattle-based digital health nonprofit developing software to improve global health in the world’s most underserved communities. Their team of passionate, innovative minds combines smartphone technology, computer vision & machine learning, and the best of cloud-based services to deliver healthcare technology solutions worldwide. The development of their projects is funded by grants from the[Bill & Melinda Gates Foundation](https://www.gatesfoundation.org/) and[Justworks](https://justworks.com/). Additional information can be found at [auderenow.org](http://auderenow.org/).
