# Supplementary material for: Identifying Enablers of Participant Engagement in Clinical Trials of Consumer Health Technologies: Qualitative Study of Influenza Home Testing
Source: J Med Internet Res. 2021 Sep 14;23(9):e26869. doi: 10.2196/26869 (PMC8479603; doi:10.2196/26869)
Supplement: Multimedia Appendix 4 [file jmir_v23i9e26869_app4.docx]

### **Table S1 Evidence Trace Table for Personal Enablers**

| **Personal Enablers** | **Representative quote for each of the identified themes in our study** |
| --- | --- |
| Digital Health Literacy | *“Usually I'll just go to my browser, my Safari browser, Google, whatever I want at the time and just type in whatever question I have, or if I have certain symptoms I'll just put that in and Google what's going on. Maybe WebMD is a big one that pops up. Just different forums. I know it'll take me to Reddit sometimes.”* |
| Motivation to advance medical research | *“Well, I just like the idea of giving research study type things. I think it’s great when more information can be found on making things easier medically and stuff.”* |
| Innovativeness | *“And I’m really interested in trying new things. I like innovative things and I was trying new things, new foods, new technology, new everything. So, I don’t know, it just enticed me. I was curious, and I enjoyed the first technology item I bought, which was, of course, a beeper and then it went to a cellphone, so having computers and the fitness watch and all those kind of things, they’re just really beneficial to me. I use them all the time, constantly.”* |
| Curiosity | *“Well, I think in part, I was curious about it, and I would say probably just curiosity.”* |
| Altruism | *“The monetary incentive or financial incentive is definitely important, but more so when something doesn’t seem to be as altruistically aligned.”* |

### **Table S2. Evidence Trace Table for Perceptional Enablers**

| **Perceptional Enablers** | **Selected participant quotes representative of the identified themes in our study** |
| --- | --- |
| Ease of Use (Nasal swab Process) | *“Simple. Very, very simple. I liked how it had the little stands that you could put your little vials in, so you don’t have to worry about, oh, if it’s going to fall over or what if it knocks over and the liquid falls out. I think it was very, very simple. I love how you guys had the packaging to get it sent back.”* |
| Ease of Use (Mobile App) | *“I thought the interface was pretty easy to use, especially for potentially some of that would be less tech-savvy. So, I thought it was a decent app. Not too complicated and served its purpose for sure.”* |
| Ease of Use (Overall trial) | *“I think it was all pretty self-explanatory. It was easy to self-manage it, and I thought it was easy to use so I really wouldn’t change anything about it.”* |
| Usefulness of flu@home | *“I just feel like people would better be able to… So, it would help you to be able to better take care of yourself. You’ll know, stay at home, don’t go to work, don’t go here. You can go to the doctor, get it taken care of, and try to keep it under control, instead of spreading germs, and more people getting sick, and going farther than what it really has to.”* |
| Usefulness of trial engagement | *“Well, I think that the testing of the flu@home and having people like me participate in it, I think it helps you guys to figure out if that’s a good way to do it, or how to adjust it to make it better…”* |
| Positive attitude toward flu@home | *“I guess just the idea of being able to do home-based checking interests me. It sounds like it has promised to me.I thought it was very cool. The idea of doing a test at home myself was really exciting... I just thought it was a super cool opportunity. I was excited to try it.”* |
| Positive attitude toward trial engagement | *“Everyone should use it. I think the trial-and-error and the feedback are really important for research staff. It’s just all the data. It’s important. I’m assuming that mine (my sample) will be used and contribute to the greater goal of good data that can help us solve this problem.”* |
| Potential to minimize doctor’s visit | “*Maybe it’s more convenient to do that(flu@home) than to try to make a doctor appointment to go in.”* |

### **Table S3. Evidence Trace Table for Situational Enablers**

| **External Enablers** | **Selected participant quotes representative of the identified themes in our study** |
| --- | --- |
| Financial incentive | *“anytime there’s an incentive to do something that goes along with finding out the same thing if you’re not feeling well, then that’s a double bonus.”* |
| Clinical Affiliation | *“When I saw that it was... The hospitals that were associated, and the university and stuff. It definitely made me trust it more and feel like it was actually a real thing. As soon as I saw who was doing the study, I told my husband, “Oh, this is definitely like legit.”* |
| Personal recommendations | *“One of my friends actually told me about it (the flu@home trial) on Facebook.” and, “My wife told me about it (the flu@home trial)”* |
| Promotion and recruitment strategies | *“I was looking up different survey things on Google and app popped up first, and I was looking into it, and this was right after I had just got over the flu. So, I was like, maybe I should try it. My boyfriend’s like, “Yeah, try it if it’s free. The app is free; just try it.” And I was like, “Okay.” And I was telling everybody, I told my boyfriend’s sister, I was like, “You guys need to try this. It’s so simple and easy. You could just do it at home. You won’t have to worry about going to the doctors.”* |
| Future recruitment strategies | *“online forum where people could volunteer to be part of studies or if there was some sort of email list that people, you could shoot out and say, “Hey, if you’re interested, you want to join this study.”* |
| Insurance status | *“I wasn’t feeling well, and as I said, I don’t have insurance at the moment, and back then, I didn’t either. So, it’s something. And I think there was an incentive for doing it, as well, so of course”* |
